# How can childhood maltreatment affect post-traumatic stress disorder in adult: Results from a composite null hypothesis perspective of mediation analysis

**DOI:** 10.3389/fpsyt.2023.1102811

**Published:** 2023-03-09

**Authors:** Haibo Xu, Zhonghe Shao, Shuo Zhang, Xin Liu, Ping Zeng

**Affiliations:** ^1^Center for Mental Health Education and Research, Xuzhou Medical University, Xuzhou, China; ^2^School of Management, Xuzhou Medical University, Xuzhou, China; ^3^Department of Epidemiology and Biostatistics, Ministry of Education Key Laboratory of Environment and Health, School of Public Health, Tongji Medical College, Huazhong University of Science and Technology, Wuhan, China; ^4^Department of Biostatistics, School of Public Health, Xuzhou Medical University, Xuzhou, China; ^5^Center for Medical Statistics and Data Analysis, Xuzhou Medical University, Xuzhou, Jiangsu, China; ^6^Key Laboratory of Human Genetics and Environmental Medicine, Xuzhou Medical University, Xuzhou, Jiangsu, China; ^7^Key Laboratory of Environment and Health, Xuzhou Medical University, Xuzhou, Jiangsu, China

**Keywords:** DNA methylation, divide-aggregate composite-null hypothesis test, gene-based mediation analysis, childhood maltreatment, psychiatric disorder

## Abstract

**Background:**

A greatly growing body of literature has revealed the mediating role of DNA methylation in the influence path from childhood maltreatment to psychiatric disorders such as post-traumatic stress disorder (PTSD) in adult. However, the statistical method is challenging and powerful mediation analyses regarding this issue are lacking.

**Methods:**

To study how the maltreatment in childhood alters long-lasting DNA methylation changes which further affect PTSD in adult, we here carried out a gene-based mediation analysis from a perspective of composite null hypothesis in the Grady Trauma Project (352 participants and 16,565 genes) with childhood maltreatment as exposure, multiple DNA methylation sites as mediators, and PTSD or its relevant scores as outcome. We effectively addressed the challenging issue of gene-based mediation analysis by taking its composite null hypothesis testing nature into consideration and fitting a weighted test statistic.

**Results:**

We discovered that childhood maltreatment could substantially affected PTSD or PTSD-related scores, and that childhood maltreatment was associated with DNA methylation which further had significant roles in PTSD and these scores. Furthermore, using the proposed mediation method, we identified multiple genes within which DNA methylation sites exhibited mediating roles in the influence path from childhood maltreatment to PTSD-relevant scores in adult, with 13 for Beck Depression Inventory and 6 for modified PTSD Symptom Scale, respectively.

**Conclusion:**

Our results have the potential to confer meaningful insights into the biological mechanism for the impact of early adverse experience on adult diseases; and our proposed mediation methods can be applied to other similar analysis settings.

## 1. Introduction

A greatly growing body of literature has revealed that adverse childhood experience is a leading neurodevelopmental risk factor, which could ultimately exhibit a long-lasting impact on many complex human disorders and diseases in adulthood such as cardiovascular dysregulation (1, 2), sleep disturbance (3), lung cancer (4), chronic metabolic diseases (5), especially neurological and psychiatric disorders (6–8). Adverse childhood event has a very generalized definition which includes any experience with the potential to be harmful to children, such as famine, sexual, and physical abuse as well as emotional and physical neglect (9). Among these, maltreatment in childhood has been attracted particular attention because it remains a major public health and social welfare problem worldwide (10, 11). For example, it is reported that about 4∼16% of children are physically abused and approximately 10% are neglected or psychologically abused in every year in high income countries and developed regions (11).

The negative consequence of childhood maltreatment not only involves significantly high morbidity of adult disorders and diseases, but also includes substantially increased risk of premature mortality. Indeed, it has been demonstrated that adults who ever had severe maltreatment experience in childhood would suffer from approximately 20 years shorter life expectancy on average compared to those without early adversity (11). Furthermore, childhood maltreatment is not only a well-established predictor of lifetime diseases such as diverse psychiatric disorders, linking with a higher occurrence of psychiatric health problems *per se*, but also associating with an earlier onset age, greater severe symptom and poorer response to psychotherapy or pharmacotherapy (12). In addition, early maltreatment experience has also been shown to have a lasting negative effect on many other important aspects of individual functions persisting from childhood into adulthood by compromising relationship quality, educational attainment, employment prospects and earnings, and physical health (12, 13). Therefore, understanding the biological mechanism of childhood maltreatment on becomes increasingly critical for developing innovative preventive and therapeutic strategies to improve health conditions.

When exploring the connection between childhood maltreatment and adult diseases, a natural question arises that why maltreatment in childhood can lead to an increased risk for negative conditions (e.g., diseases/disorders) in adulthood even many years after such an exposure itself has ceased (12). There are several possibly reasonable interpretations to answer this question and to explain the enduring influence of childhood maltreatment on adult diseases, among which the significant role of epigenetics has emerged as a promising mechanism underlying the biological sequelae of maltreatment in childhood on adverse outcome in adult (9, 14–18). Biologically, epigenetic regulation enables the body to respond to the influence of environmental factors by altering gene expression level through chemical modifications which modulate chromatin structure and/or DNA accessibility without inducing alteration to the DNA sequence. In the literature, DNA methylation is one of the most frequently investigated epigenetic regulation processes and involves the addition of a methyl group at sites in the DNA where a cytosine nucleotide occurs next to a guanine nucleotide (CpG dinucleotides).

Previous studies have established the relationship between childhood maltreatment, DNA methylation and various complex diseases/disorders. For example, it was shown that early life stress was significantly associated with DNA methylation in rodents and that low levels of maternal care (licking, grooming, and arched-back nursing) were related to greater level of methylation of the glucocorticoid receptor gene in offspring (14, 15). Lutz and Turecki showed that early life experience (e.g., childhood abuse) can modify lifelong stressful activities (e.g., psychopathological disorders) through DNA methylation regulation (19). Klengel et al. (20) found that exposure to stress would cause long-term changes in DNA methylation, which may be in turn related to the pathophysiology of psychiatric disorders such as post-traumatic stress disorder (PTSD) and depression. Several recent systematic reviews offered a comprehensive description about the connection between early maltreatment experience, altered patterns of DNA methylation and complex human diseases (9, 12, 21). Statistically, the evidence above suggests that an intermediary model can help understand how childhood maltreatment alters long-lasting changes of DNA methylation, which further affects psychiatric disorders (22–27).

However, the mechanisms regarding how childhood maltreatment possibly influences diseases remain largely elusive. Methodologically, this motivates us to consider DNA methylation as a critical causal mediator of childhood maltreatment on the development of adult diseases using mediation analysis models. As a result, the essential requirement of mediation analysis arises when we are interested in potential mechanism between an exposure (e.g., childhood maltreatment) and an outcome (e.g., psychiatric disorder) and long to acquire in-depth insight into such understanding. Formally, a mediating variable, also known as mediator, is defined as an intermediate variable (e.g., DNA methylation) in the causal sequence that connects the exposure with the outcome (28). From a statistical perspective, mediation analysis can be applied to investigate the interplay between childhood maltreatment, DNA methylation and disorder (28–35).

Traditionally, single CpG site mediation analysis is conducted to identify methylations with mediating role; however, as the number of CpG sites can be much larger than sample size, mediation analysis focusing on one mediator at a time has become increasingly challenging and often infeasible to directly apply them towards the large and complex data collected in an epigenetic study (35, 36). Furthermore, the following three facts motivate us to implement gene-centric mediation analysis in the present work. First, methylation signals within a gene are often correlated with each other, sometimes quite strongly. For example, methylations on proximal CpG sites are generally similar to each other and genes in the same pathway often show coordinated co-expression pattern (37). Such correlation, if not correctly handled, would result in false discoveries in the traditional single CpG site mediation analysis. Second, as genes are important functional units in living organisms, gene-based mediation analysis offers more biologically meaningful results. Third, a gene likely contains several mediation signals, single CpG site mediation analysis is thus conservative and does not fare well for large-scale multiple testing tasks (38–41); in contrast, analyzing the mediation effect of the whole gene rather than focusing on individual methylation site is generally more powerful (34).

**FIGURE 1:**
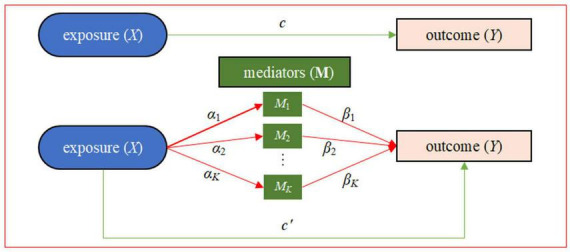
Statistical framework of the gene-centric mediation analysis with one exposure *X* (e.g., childhood maltreatment), multiple DNA methylation mediators M, and one outcome *Y* (e.g., PTSD). Here, α = (α_1_, …, *α_*K*_*) is the vector of effect sizes of the exposure on *K* mediators in the exposure-mediator model, **β** = (β_1_, …, *β_*K*_*) is the vector of effect sizes of the *K* mediators on the outcome in the mediator-outcome model, *c*′ is the direct effect conditional on the mediators, and *c* is the total exposure on outcome effect. There are two types of effects from *X* to *Y*: the direct effect of *X* on *Y* (i.e., *c*′) and the indirect effect of *X* on *Y via* the intermediate variables M. The indirect effect, also known as mediation effect denoted by **αβ**, represents the amount of mediation coming from two sources: the effect from *X* to M (i.e., **α**) and the effect from M to *Y* (i.e., **β**).

In this study, with relevant data available from the Grady Trauma Project (GTP) as an illustrative example (8, 42), we intend to carry out a mediation analysis using composite null hypothesis to examine the mediating role of DNA methylation on the signaling pathway from childhood maltreatment to psychiatric disorders in adult, where the two path association tests could be implemented through an equivalent manner of variance-component based score tests under the context of mixed models. More specifically, we would implement the gene-centric mediation analysis to simultaneously model a group of DNA methylation CpG sites as mediators and explore their mediating role in the impact of childhood maltreatment on psychiatric disorders (Figure 1).

To effectively detect the existence of mediation effect with high-dimensional mediators, we first employ the variance component-based score test in a linear mixed model *via* an inverse transformation to assess the association between childhood maltreatment and a group of DNA methylation CpG sites, and then test for the effect of DNA methylation on the disorder of focus while adjusting for the direct influence of childhood maltreatment within the framework of linear mixed-effects model (43–46). We finally determine whether the mediation effect of DNA methylation exists by following the main idea proposed in Liu et al. (41), which can lead to desirable type I error rate control and higher power compared to existing mediation effect test methods.

## 2. Materials and methods

### 2.1. Data description of the Grady Trauma Project

The GTP was a cross-sectional study dedicated to investigate the role of environmental and genetic risk factors in the development of stress-related psychiatric disorders, such as PTSD, depressive disorder, and anxiety disorder, that confer a heavy burden on public health worldwide (8, 47). We downloaded datasets of various types of depression/stress scores (e.g., current stress score and cumulative life stress score), baseline features (e.g., sex and age) and DNA methylation for African-American participants with PTSD from GTP (the GEO accession number: GSE72680).

The exposure of our interest is childhood maltreatment including emotional abuse, physical abuse, sexual abuse, emotional neglect, and physical neglect, which was measured using the Childhood Trauma Questionnaire (48) and further dichotomized in terms of the moderate to extreme criteria. Current PTSD symptoms and depression symptoms were assessed with the modified PTSD Symptom Scale (PSS) (49) and the Beck Depression Inventory (BDI) (50), respectively. Following prior work (51), in our analysis we defined PTSD cases with comorbid current PTSD and depression symptoms (PTSD&Dep) as having PSS score ≥14 and BDI score ≥14, and defined controls with no current PTSD and no depressive symptoms as having PSS score ≤7 and BDI score ≤7 despite being exposed to trauma. Other continuous outcomes of interest include distinct types of depression or stress scores (Table 1).

**TABLE 1:** Descriptive statistics for 352 participants in the GTP data after quality control.

Variables	PTSD/Depression case (*n* = 191)	Control (*n* = 161)	Total (*n* = 352)
**Sex (*n*, %)**			
Male	58 (33.0)	36 (25.4)	94 (29.6)
Female	118 (67.0)	106 (74.6)	224 (70.4)
**Childhood maltreatment (*n*, %)**			
No	87 (49.4)	92 (64.8)	179 (56.3)
Yes	89 (50.6)	50 (35.2)	139 (43.7)
BMI ($\bar{x}$± SD)	32.1 ± 8.6	32.3 ± 8.2	32.2 ± 8.4
Age ($\bar{x}$± SD)	41.9 ± 12.9	41.0 ± 13.5	41.5 ± 13.2
Cumulative life stress score ($\bar{x}$± SD)	12 ± 3.9	9.7 ± 3.5	11.02 ± 3.9
Network life stress score ($\bar{x}$± SD)	3.8 ± 1.6	3.1 ± 1.4	3.5 ± 1.6
Personal life stress score ($\bar{x}$± SD)	8.2 ± 2.9	6.7 ± 2.7	7.5 ± 2.9
Current stress score ($\bar{x}$± SD)	5.5 ± 2.9	4.0 ± 2.4	4.8 ± 2.8
Beck score ($\bar{x}$± SD)	19.6 ± 12.3	9.5 ± 8.1	15.6 ± 11.9
PTSD score ($\bar{x}$± SD)	18.7 ± 12.4	8.0 ± 8.9	14.0 ± 12.3

Beck score, Beck depression inventory total score; PTSD score, post-traumatic stress disorder symptom scale total score; childhood maltreatment, childhood maltreatment score that was divided into moderate to extreme.

For quality control we filtered out those already receiving PTSD-relevant treatments (such as treatments for depression, bipolar disorder, PTSD, and anxiety disorder) since the treatments might affect DNA methylation changes which can complicate the mediation effect. More details about the GTP study can be found elsewhere (8, 42).

DNA methylation levels of these GTP subjects were measured in peripheral blood using the Illumina Infinium Human Methylation 450K BeadChip. Batch effects and other potential latent sources of variation [e.g., environmental variables (52) and genetic variation (53, 54)] were removed by using R package “sva” (version 3.35.2) (55). We removed non-CpG sites (probes with ch labels) and CpG sites located on sex chromosomes, and excluded cross-reactive probes as suggested in Chen et al. (56). After the data processing, 264,564 DNA methylation CpG sites were retained. Furthermore, the beta value for each DNA methylation at a CpG site was logit-transformed to generate the M-value for each locus because, unlike the beta value, the M-value is not bounded between zero and one and is thus more valid for statistical modeling (57).

We adjusted for the confounding effects of age, sex, body mass index (BMI) when examining the association between childhood maltreatment and DNA methylation as well as the association between DNA methylation and PTSD or other score outcomes. In addition, cell type was the single most important known factor in determining DNA methylation profiles (58) and some differences in cell types might significantly confound results from DNA methylation analyses (59, 60); we therefore included blood cell type (i.e., CD4T, CD8T, Mono, B cell, and NK) proportions as covariates, which was directly obtained by the laboratory file of the data.

### 2.2. Statistical model for our gene-centric mediation analysis

We treat childhood maltreatment as the exposure and PTSD (or score) as the outcome, and apply an epigenetic mediation framework to investigate how the maltreatment in childhood alters long-lasting DNA methylation changes, which in turn affects adult psychiatric disorders such as PTSD (Figure 1). More specifically, we would carry out a gene-centric mediation analysis (34, 35, 40), where each mediation effect test includes one exposure *X*, multiple methylation mediators **M** which are *per se* likely high-dimensional, and one outcome *Y* that may be continuous or binary for *n* individuals. According to the gene annotation mapping file we define the set of DNA methylation CpG sites for each gene as those located within the entire gene body and 500 bps upstream of the transcription start site (TSS) so that the promoter can be included (61, 62). A total of 16,295 genes were analyzed, with the median of the number of DNA methylation CpG sites per gene equal to 16.

In the mediation analysis the influence of the exposure *X* on the outcome *Y* stands for the direct effect (denoted by *c*′ in Figure 1), and the influence of *X* on **M** and subsequently **M** on *Y* for the indirect effect which is also called the mediation effect (denoted by αβ in Figure 1). If *X* affects *Y* only through **M** (i.e., *c*′ is zero), such association is referred to as full mediation; otherwise, it is referred to as partial mediation (i.e., *c*′ is non-zero) (35, 63). Our objective is to assess whether the causal effect of *X* on *Y* is mediated *via* **M**. Note that, traditionally, the presence of a significant total effect (i.e., *c* ≠ 0 in the outcome-exposure model) is the prerequisite for the mediation effect test (29). However, in practice the situation in which the total effect is nonsignificant but a substantial mediation effect remains is not uncommon; for example, the direct effect is opposite in sign to the indirect effect and has a similar size in magnitude, which is referred to as suppression or inconsistent mediation (64–68). Therefore, following prior studies (66, 67), in the present work we always carry out our mediation effect test regardless of whether there exists a substantial total effect of the exposure on.

For each gene under analysis, we first apply an inverse regression as done in Djordjilović et al. (69) and treat the impacts of CpG sites (mediators) on childhood maltreatment (exposure) as random effects; thus the associations between childhood maltreatment and methylation in the exposure-mediator model can be derived by applying a variance component test in the linear mixed model. In the next step, we use the same strategy to utilize a mixed model and assess the association between methylation and PTSD (or scores) in the mediator-outcome model by Davies’ method *via* the SKAT package (43, 70). Finally, a joint significance test is conducted to evaluate the significance of the mediation effects using a Benjamini–Hochberg false discovery rate (FDR) correction for multiple testing. To his aim, we here follow the main idea of divide-aggregate composite-null test (DACT) proposed in Liu et al. (41); to formally obtain the *P*-value of mediation effect, we rely on a modified test statistic and utilize the Efron empirical null framework to estimate the proportions of the three sub-null hypotheses across whole genome mediators. The pipeline of this mediation analysis is described in detail in Supplementary material.

## 3. Results

### 3.1. Descriptive statistics for the GTP data

A total of 191 cases with the symptom of comorbid current PTSD/depression and 161 controls were finally reserved for further analysis. Between-group differences in clinical features of all the remaining participants are summarized in Table 1. The position of DNA methylation CpG sites of all the 352 participants were determined by gene symbols, and a total of 16,565 unique genes were obtained.

### 3.2. Association from childhood maltreatment to PTSD or scores, from maltreatment to DNA methylation, and from DNA methylation to PTSD or scores

We first examined the association between childhood maltreatment and PTSD or other PTSD-related score outcomes (i.e., *H*_0_: *c* = 0). The results for the total effect are shown in Table 2. It was observed that the maltreatment in childhood substantially affected the development of PTSD or increased PTSD-relevant scores after adjusting for the multiple-test issue at the FDR level of 0.05. For example, the experience of extreme maltreatment in childhood would significantly result in higher risk [odds ratio = 2.16, 95% confidence intervals (CIs) 1.35∼3.46) of occurring PTSD in adult (*P* = 1.65 × 10^–3^), lead to an increase of 6.55 units (95% CIs 3.76∼9.33) in the BDI total score (*P* = 7.25 × 10^–6^) or an elevation of 1.86 units (95% CIs 1.19∼2.52) in the current stress score (*P* = 1.07 × 10^–7^).

**TABLE 2:** Results of the gene-centric mediation analysis with childhood maltreatment as the exposure, DNA methylation CpG sites located within a gene as mediators, and PTSD and six PTSD-relevant scores as outcomes.

Outcomes	Total effect		Count of significant association		
	***c* (SE; CIs)**	***P***	**α**	**β**	**αβ**
Beck score	6.55 (1.43; 3.76–9.33)	7.25 × 10^–6^	670	631	13
Current stress score	1.86 (0.34; 1.19–2.52)	1.07 × 10^–7^	888	640	6
Cumulative life stress score	2.63 (0.48; 1.69–3.56)	9.28 × 10^–8^	1,091	476	0
Network life stress score	0.74 (0.19; 0.37–1.11)	9.98 × 10^–5^	1,392	349	0
Personal life stress	1.78 (0.34; 1.12–2.44)	4.50 × 10^–7^	1,155	513	0
PTSD&Dep score (continuous)	9.30 (1.34; 6.69–11.91)	2.78 × 10^–11^	1,168	1,106	0
PTSD&Dep (binary)	0.77 (0.24; 0.30–1.24)	1.65 × 10^–3^	1,032	1,996	0

We next evaluated the association between childhood maltreatment and a group of DNA methylation CpG sites (i.e., *H*_0_: α = 0) and the association between a set of DNA methylation and PTSD (and various score outcomes) for each gene (i.e., *H*_0_: β = 0). As a result, it was shown that the maltreatment in childhood possibly affected many promising DNA methylation CpG sites across these PTSD-relevant outcomes, with the number of significant associations ranging from 670 for the BDI total score to 1,392 for the network life stress score (*P* < 0.05). On the other hand, after the adjustment of the direct effect of maltreatment, we identified many DNA methylation CpG sites associated with PTSD or other PTSD-related score outcomes, with the number of promising association signals ranging from 349 for the network life stress score to 1,996 for PTSD (*P* < 0.05).

### 3.3. DNA methylation CpG sites with mediating effects

Finally, we applied DACT to detect DNA methylation CpG sites that may exert mediating effects on the path from the maltreatment in childhood to PTSD-relevant outcomes in adult. We found that there were 13 and 6 genes with methylation CpG sites having mediation effects for the BDI total score and the current stress score (FDR <0.05), respectively. In addition, although methylation CpG sites in these genes exhibited mediating roles, only a few sites were significantly differentially methylated between the cases and controls (FDR <0.05). We show more detailed information regarding these DNA methylation CpG sites in Table 3.

**TABLE 3:** Located genes of DNA methylation CpG sites with mediation effects for the Beck depression inventory total score and the current stress score.

Outcome	Gene (methylation sites located within a gene)	Chr	Lower	Upper	*m* (m*)	α^$^	*P_α_*	β	*P_β_*	*P_αβ_*	FDR
Beck score	*RNF144A*	2	7,056,074	7,237,581	27 (0)	+	8.73 × 10^–3^	+	7.55 × 10^–3^	1.04 × 10^–6^	8.59 × 10^–3^
	*TMPRSS4*	11	117,939,669	117,964,271	9 (0)	+	2.83 × 10^–2^	+	3.17 × 10^–3^	1.05 × 10^–5^	1.80 × 10^–2^
	*PPM1L*	3	160,472,555	160,746,047	27 (3)	+	2.09 × 10^–2^	+	2.38 × 10^–2^	3.64 × 10^–5^	4.64 × 10^–2^
	*SAMD9*	7	92,626,635	92,748,364	15 (0)	+	9.12 × 10^–3^	−	2.24 × 10^–2^	1.09 × 10^–5^	1.80 × 10^–2^
	*CNRIP1*	2	68,520,245	68,557,961	18 (1)	+	2.52 × 10^–2^	+	1.11 × 10^–3^	5.57 × 10^–6^	1.45 × 10^–2^
	*NHLRC4*	16	615,708	619,456	2 (0)	+	5.90 × 10^–3^	+	2.33 × 10^–2^	8.35 × 10^–6^	1.73 × 10^–2^
	*RNF208*	9	140,108,140	140,117,444	10 (1)	+	2.08 × 10^–2^	+	5.16 × 10^–3^	5.38 × 10^–6^	1.45 × 10^–2^
	*MYO7A*	11	76,838,497	76,927,760	35 (0)	+	1.63 × 10^–2^	+	4.06 × 10^–3^	2.26 × 10^–6^	1.25 × 10^–2^
	*ABAT*	16	8,767,705	8,862,654	32 (8)	+	4.50 × 10^–3^	+	3.70 × 10^–2^	2.86 × 10^–5^	3.95 × 10^–2^
	*APOC1*	19	45,416,368	45,422,542	6 (1)	+	3.58 × 10^–2^	+	1.16 × 10^–3^	1.84 × 10^–5^	2.78 × 10^–2^
	*NPS*	10	129,346,390	129,379,354	8 (0)	−	9.46 × 10^–3^	−	1.73 × 10^–2^	6.12 × 10^–6^	1.45 × 10^–2^
	*SLC25A23*	19	6,459,034	6,460,275	9 (2)	+	1.01 × 10^–2^	+	1.12 × 10^–4^	1.89 × 10^–7^	3.13 × 10^–3^
	*CLC*	19	40,227,948	40,229,767	6 (0)	−	2.60 × 10^–3^	+	2.35 × 10^–2^	5.63 × 10^–6^	1.45 × 10^–2^
Current stress score	*STRAP*	12	15,990,182	16,049,852	16 (0)	+	5.69 × 10^–3^	+	1.30 × 10^–4^	2.72 × 10^–7^	4.50 × 10^–3^
	*TLL1*	4	166,793,788	167,022,416	18 (0)	−	2.11 × 10^–3^	+	4.25 × 10^–3^	7.57 × 10^–7^	6.27 × 10^–3^
	*SNAP91*	6	84,415,073	84,422,334	17 (0)	−	8.00 × 10^–3^	+	3.29 × 10^–3^	2.71 × 10^–6^	1.50 × 10^–2^
	*HIST1H2AA*	6	25,726,436	25,727,292	2 (0)	+	1.06 × 10^–3^	+	1.39 × 10^–2^	1.22 × 10^–5^	3.37 × 10^–2^
	*HIST1H2BA*	6	25,726,436	25,727,292	5 (0)	+	1.06 × 10^–3^	+	1.39 × 10^–2^	1.22 × 10^–5^	3.37 × 10^–2^
	*KRTAP9-2*	17	39,382,246	39,383,027	4 (0)	−	4.28 × 10^–3^	−	8.37 × 10^–3^	5.70 × 10^–6^	2.36 × 10^–2^

The name of the gene within which these disordered methylation sites were located was listed in the second column; Chr, lower and upper in the next three columns denoted the chromosome and the position of the gene (hg19 given by UCSC Xena); *m* stands for the number of DNA methylation CpG sites per gene; note that, after the data processing, 264,564 DNA methylation CpG sites and 16,295 genes were analyzed, resulting in a median of 16 CpG sites per gene; m* denotes the number of significantly differential methylated sites between the cases and controls (FDR <0.05); ^[dollar]^indicates the effect direction after aggregating each element of α or β.

## 4. Discussion

A great deal of literature has revealed the mediating role of epigenetic alteration such as DNA methylation standing on the path from early adverse experience (e.g., childhood maltreatment) to adult psychotic disorders (9, 12, 14–18). However, to our knowledge, there are currently only few studies which formally investigate this important issue under the hypothesis testing framework of composite null hypothesis mediation analysis. The present work is exactly among the first to explore whether DNA methylation CpG sites have a substantial mediating effect from a contemporary mediation perspective of composite null hypothesis testing. Previous work has already demonstrated that considering the composite null nature in mediation effect test holds the key to improve power for identifying active mediators in mediation analysis (34, 35, 38–41, 71).

In the present study, we applied a recently proposed novel method called DACT to determinate which DNA methylation CpG sites may play the mediating role in the connection between childhood maltreatment and PTSD or PTSD-related scores. The advantage of DACT is that it only takes two sets of *P*-values as input, can thus be scalable to studies with large scale sample size in mediation analysis (41). In addition, DACT can borrow the useful information of all DNA methylation mediators across the whole genome to estimate some necessary parameters. Note that, it has been shown that, compared with other composite-null based mediation effect test methods such as the JS-comp which circumvents estimating the proportion parameters of composite null sub-hypotheses under some strong modeling assumptions (40, 72), considering study-specific scenarios and estimating these proportion parameters are crucial to generate well-calibrated type I error control in mediation analysis (34, 35, 41).

In addition, unlike prior work which considered a single DNA methylation CpG site in each time (36), we instead analyzed a group of DNA methylation CpG sites located within their nearby gene jointly when detecting active methylation mediators. As demonstrated in previous work (34), the number of DNA methylation CpG sites can be much greater than sample size; thus, focusing on one mediator at a time is not efficiently powerful enough to handle thousands of mediators. The strength of this gene-centric mediation analysis is that it has the potential to further enhance the power by aggravating multiple weak association signals. In the meantime, this gene-centric mediation effect method also challenges the traditional single mediator mediation analysis (40). We effectively addressed this challenging task by leveraging a random-effect model in which we assumed the effects of the exposure on the mediators and the effects of multiple mediators on the outcome followed a normal distribution with an unknown variance parameter. Therefore, the two path association tests in our mediation analysis can be implemented by an equivalent manner of variance-component based score tests under the context of mixed models. It has been demonstrated that the variance component test is often more powerful compared to its counterpart such as multivariate Wald test (43, 44, 46, 73–76).

As a result, using DACT with two *P*-values available from variance component tests, we discovered a set of DNA methylation within some genes having promising mediation effect. Many of these genes were previously demonstrated to be associated with psychiatric disorders such as stress, depression, schizophrenia, and PTSD. Note that, psychiatric disorders are highly correlated in genetic foundation and clinical manifestation (77–82). For example, *PPM1L*, encoding protein phosphatase, was detected to be responsible for the regulation of stress-activated protein kinase signaling cascade and apoptosis (83, 84). It is worth noting that this gene also identified by a previous study (33). *CNRIP1*, encoding the cannabinoid receptor interacting protein 1, is an intracellular protein that interacts with the C-terminal tail of *CB1R* and regulates its intrinsic activity. Aberrant *CNRIP1* DNA promoter methylation was observed in post-mortem in human patients with schizophrenia (85), and decreased methylation of the *CNRIP1* DNA promoter was discovered in the ventral hippocampus of a rodent model of schizophrenia susceptibility (86). Recent work showed that ventral hippocampal overexpression of *CNRIP1* would lead to a schizophrenia-like phenotype in the rat (87). In addition, *CNRIP1* was a regulator involved in the neural development (88). *SLC25A23* encodes the human APC2 (ATP-Mg/Pi carrier), which is a part of Ca2+ sensitive mitochondrial carriers; and *SLC25A23* mediates the influx of Ca2+ and regulates ATP levels in neurons (89). The altered *SLC25* family gene expression was revealed to be a marker of mitochondrial dysfunction in brain regions under experimental mixed anxiety/depression-like disorder (90). Moreover, *SLC25A23* was identified as a candidate biomarker gene that can improve low mood state of major depressive disorder (MDD) (91); a recent study further confirmed this finding and demonstrated that up-regulation of *SLC25A23* might be associated with MDD (92). As another example, a genome-wide association study showed that a genetic variant mapping to the first intron of *TLL1* was strongly related to PTSD, implying that *TLL1* might be a susceptibility gene for this disorder (93). Additionally, *SNAP91*, encoding the synaptosome-associated protein 91, was identified to be a risk gene of schizophrenia (94, 95).

Besides the direct evidence described above, prior studies also offered indirect evidence supporting the association for some of these gene with brain-related diseases such as neurodegenerative diseases. For instance, mutations of *MYO7A* were found to be responsible for Usher syndrome, a brain-related disease (96). *ABAT*, a key enzyme responsible for catabolism of principal inhibitory neurotransmitter γ-aminobutyric acid (GABA), exhibited the connection between GABA metabolism and nucleoside metabolism and played a key role in developing neurometabolic disorders such as mtDNA depletion syndrome (97). In addition, *APOC1* encodes a member of the apolipoprotein C1 family, which plays a well-established role in the transport and metabolism of lipids within the central nervous system and is critical for healthy brain function. The importance of apolipoproteins in brain physiology is also highlighted by many genetic studies, where apolipoprotein gene polymorphisms have been identified as risk factors for several neurological diseases (98). In addition, it is shown that the *APOC1* insertion allele, in combination with APOE ε4, likely served as a potential risk factor for developing Alzheimer’s disease (99).

Finally, our study is not without limitations. First, we assumed that all DNA methylation CpG sites were affected by childhood maltreatment and had influences on PTSD-relevant outcomes. This assumption might be not true as it was likely only a fraction of methylation with substantial effects. Consequently, under this scenario a sparse relationship was more suitable. Second, because PTSD is a brain-relevant disorder and it was impossible and ethically unreasonable for the availability of brain tissues from individuals alive, in this work the analyzed DNA methylation had to be measured in peripheral blood, which might not fully reflect DNA methylation alteration in brain tissues. Third, although they might be considerably important, some other covariates (e.g., tobacco smoking, alcohol drinking, cocaine use, heroin use, and marijuana use) were simply ignored in accordance with previous study (36, 100) as these variables had relatively high missing values. Variation in DNA methylation is strongly associated with lifestyle factors such as smoking behavior (101–103); one possible solution to covariates with high missing values is to develop a methylation score to efficiently predict smoking status (104–107). However, if we attempt to apply this predictive approach to impute missing data, to avoid model overfitting we must look at a random split of the data into two non-overlapping samples—one for prediction and the other for mediation analysis, which would certainly lead to reduced power in mediation effect test. Fourth, only one PTSD dataset was analyzed, no external datasets were used for validating our discovered methylation sites with mediating effects. In addition, further longitudinal data that can provide time-span facts for discovered DNA methylation CpG sites is particularly warranted.

## 5. Conclusion

In conclusion, by leveraging novel statistical methods we identified multiple promising DNA methylation sites that had mediating roles in the influence path from childhood maltreatment to PTSD-relevant outcomes in adult. Our results have the potential to confer meaningful insights into the biological mechanism for the impact of early adverse experience on adult diseases; and our proposed mediation methods can be applied to other similar analysis settings.

## Data availability statement

The data presented in the study are deposited in the GTP repository, accession number GSE72680. The data can be found at: https://www.ncbi.nlm.nih.gov/geo/query/acc.cgi?acc=GSE72680.

## Author contributions

PZ and HX contributed to conception of the study. PZ and ZS obtained the data, cleared up the datasets, and performed the data analyses. PZ, SZ, HX, XL, and ZS interpreted the results of the data analyses, and drafted and revised the manuscript. All authors contributed to the article and approved the submitted version.
