# Supplementary material for: How can childhood maltreatment affect post-traumatic stress disorder in adult: Results from a composite null hypothesis perspective of mediation analysis
Source: Front Psychiatry. 2023 Mar 9;14:1102811. doi: 10.3389/fpsyt.2023.1102811 (PMC10033829; doi:10.3389/fpsyt.2023.1102811)
Supplement: Supplementary file 1 [file Data_Sheet_1.docx]

Supplementary Material

# Supplemental Methods

## Evaluating the association between childhood maltreatment and methylation in the exposure-mediator model

First, let the sample size be *n* and ***Z*** be a set of covariates needed to control for. We aim to examine whether the exposure ***X*** is substantially associated with these methylation mediators in the exposure-mediator model. To this aim, we employ a multivariate linear model to characterize the relationship between childhood maltreatment and multiple CpG sites for all incorporated PTSD subjects

$$M_{k} = X\alpha_{k} + Zw_{1} + \varepsilon_{M}^{k}, k = 1, ...\text{，} K$$

where ***M***=(*M*_1_, …, *M_K_*) is a *K*-dimensional vector of methylation mediators, ***α***=(*α*_1_, …, *α_K_*) is the effect size of childhood maltreatment on the methylation mediators, *K* is the total number of DNA methylation CpG sites belonging to the gene of focus; ***w***_1_*_k_* denotes the vector of effect sizes of covariates in the exposure-mediator model; and ***ε****_M_*=(*$\varepsilon_{M}^{1}$*, …, *$\varepsilon_{M}^{K}$*) is a multivariate residual vector with mean zero and covariance matrix **Σ**. We examine *H*_0_: ***α***=0 in the above model.

In order to avoid a challenging task of estimating **Σ** and making any assumptions about its structure, we leverage the classical Frisch-Waugh-Lovell theorem ([Lovell, 1963](#_ENREF_11), [Frisch and Waugh, 1933](#_ENREF_8)) and assess an equivalent hypothesis test $H_{0}^{*}: \tilde{\boldsymbol{\alpha}}$=0 by constructing an inverse regression as done in ([Djordjilović et al., 2019](#_ENREF_5))

$$X = M\tilde{\alpha} + Z\tilde{w}_{1} + \varepsilon_{X}$$

One commonly-used strategy is to treat $\tilde{\boldsymbol{\alpha}}$ as fixed effects and apply the classical score test (or multivariate Wald test) for hypothesis testing. However, as the number of CpG sites (i.e., *K*) located within a gene might be very large and highly correlated with each other, the fixed-effects test method would result in the reduction in degrees of freedom and are thus underpowered ([Qiao et al., 2022](#_ENREF_13), [Wu et al., 2011](#_ENREF_15), [Qu et al., 2013](#_ENREF_14), [Zeng et al., 2014](#_ENREF_16)). Alternatively, we assume each $\tilde{\alpha}_{k}$ follows a normal distribution with zero mean and a unknown variance *N*(0, τ_1_); then, testing for the null hypothesis$H_{0}^{*}: \tilde{\boldsymbol{\alpha}}$=0 equivalently becomes examining *H*_0_: τ_1_=0, which is a variance component test in the linear mixed model. The corresponding score test statistic is given as

$$Q_{M} = \sum_{k = 1}^{K} \left\{\sum_{i = 1}^{n} \left[M_{ik}(X_{i} - Z_{i}\hat{\tilde{w}}_{1})\right]^{2}\right\}$$

where $\hat{\tilde{w}}_{1}$ is the estimate of $\tilde{w}_{1}$ under the null model. Under $H_{0}^{*}$, *Q_M_* follows a mixture of chi-square distribution and the *P*-value is obtained by the method of Davies ([Davies, 1980](#_ENREF_4), [Wu et al., 2011](#_ENREF_15)). We implement this test via the SKAT package ([Wu et al., 2011](#_ENREF_15)). Afterwards, we obtain a *P* value, denoted by *P****_α_***, for *Q_M_* as the evidence for assessing the association between childhood maltreatment and methylation.

## Assessing the association between methylation and PTSD (or scores) in the mediator-outcome model

To evaluate the association between methylation and PTSD (or scores) in the mediator-outcome model while adjusting for the direct effect of childhood maltreatment, we construct the following mixed model

$$g\left\{E(Y)\right\} = \sum_{k = 1}^{K} M_{k}\beta_{k} + Xc' + Zw_{2}$$

where *g*(·) denotes the identify link function for a continuous outcome (e.g., stress score) or the logit link function for a binary outcome (e.g., PTSD), ***w***_2_ denote the vector of effect sizes of covariates in the exposure-mediator model. With the same reason stated above, we here assume each *β_k_* follows a normal distribution *N*(0, τ_2_). Based on this condition, we examine the null hypothesis *H*_0_: τ_2_=0 (or equivalently *H*_0_: ***β***=(*β*_1_, …, *β_K_*)=0) by utilizing the variance-component based score test with a linear kernel ([Wu et al., 2011](#_ENREF_15))

$$Q_{Y} = \sum_{k = 1}^{K} \left\{\sum_{i = 1}^{n} \left[M_{ik}(Y_{i}-X\hat{c'}-Z_{i}\hat{w}_{2})\right]^{2}\right\}$$

where $\hat{c'}$ and $\hat{w}_{2}$ are the estimates of *c′* and ***w***_2_ under the null model (i.e.,$g[E(Y)] = Xc' + Zw_{2}$), respectively. Again, under *H*_0_: τ_2_=0, *Q_Y_* follows a mixture of chi-square distribution and the *P* value is obtained by Davies’ method via the SKAT package ([Davies, 1980](#_ENREF_4), [Wu et al., 2011](#_ENREF_15)). Afterwards, we obtain a *P* value, denoted by *P****_β_***, for *Q_Y_* as the evidence for assessing the association between methylation and the outcome.

## DACT method for examining the significance of mediation effect

Finally, to verify whether these methylations have substantial mediation effect on the path from childhood maltreatment to PTSD-relevant outcome, we test for the joint null hypothesis *H*_0_: ***αβ***=0 (or equivalently, *H*_0_: τ_1_τ_2_=0), which can be divided into three composite null sub-hypotheses

$$H_{0} = \left\{\begin{matrix} H_{10} : \alpha\neq0 (\text{τ}_{1} \neq0 ) \text{and} \beta\text{=} 0 (\text{τ}_{2} = 0 ) \\ H_{01} : \alpha= 0 (\text{τ}_{1} = 0 ) \text{and} \beta\neq0 (\text{τ}_{2} \neq0 ) \\ H_{00} : \alpha= 0 (\text{τ}_{1} = 0 ) \text{and} \beta\text{=} 0 (\text{τ}_{2} = 0 ) \end{matrix}\right.$$

Naturally, we can select the maximum of *P****_α_*** and *P****_β_*** (denoted by *P*_max_) for examining the existence of mediating effect, which is therefore referred to as the maximum *P*-value method or joint significance test (JST) in the mediation analysis literature ([Barfield et al., 2017](#_ENREF_1), [MacKinnon et al., 2002](#_ENREF_12)). Under certain regularity conditions, JST is a level-*α* test, suggesting that the type I error of JST is guaranteed at most *α* once the rejection decision for *H*_0_ is made ([Berger and Hsu, 1996](#_ENREF_2)). However, it is unclear how to achieve these conditions when implementing JST in our gene-centric mediation effect test.

It is easy to see that JST essentially uses the zero-one uniform distribution as its null distribution. As revealed by prior studies ([Dai et al., 2022](#_ENREF_3), [Barfield et al., 2017](#_ENREF_1), [MacKinnon et al., 2002](#_ENREF_12), [Liu et al., 2021](#_ENREF_10)), JST would be overly conservative especially when *H*_00_ holds, which is particularly true in the genome-wide mediation analysis. To correct the conservativeness of JST, we here follow the main idea of divide-aggregate composite-null test (DACT) proposed in ([Liu et al., 2021](#_ENREF_10)) by relying on a modified test statistic and directly estimating the proportions of the three sub-null hypotheses across whole genome mediators. Intuitively, Under *H*_01_, if the effects of mediators (e.g., DNA methylation) on the outcome (e.g., PTSD) are non-zero (i.e., ***β***≠0), then we only need to test *H*_01_: ***α***=0 and applies *P****_α_*** for assessing the significance of meditation effect. Under *H*_10_, if the effects of exposure (e.g., childhood maltreatment) on mediators are non-zero (i.e., ***α***≠0), then we only need to evaluate test *H*_10_: ***β***=0 and uses *P****_β_*** for evaluating meditation effect. Notably, under *H*_00_, *P*_max_ follows Beta(2, 1) ([Liu et al., 2021](#_ENREF_10)); therefore, $P_{\max}^{2}$, rather than *P*_max_ in JST, follows a uniform null distribution. Taking these together, DACT generates the *P* value for testing *H*_0_: ***αβ***=0 as a weighted summation of *P* values under the three sub-null hypotheses

$$P_{\text{DACT}} = w_{01}P_{\alpha} + w_{10}P_{\beta} + w_{00}P_{\text{max}}^{2}$$

where the weights are given as

$$\begin{matrix} w_{01} = (1 \text{-} \text{π}_{\beta0})\text{π}_{\alpha0}/\varpi\\ w_{10} = (1 \text{-} \text{π}_{\alpha0})\text{π}_{\beta0}/\varpi\\ w_{00} = (1 \text{-} \text{π}_{\alpha0})(1 \text{-} \text{π}_{\beta0})/\varpi\\ \varpi= \text{π}_{\alpha0}(1 \text{-} \text{π}_{\beta0}) + (1 \text{-} \text{π}_{\alpha0})\text{π}_{\beta0} + (1 \text{-} \text{π}_{\alpha0})(1 \text{-} \text{π}_{\beta0}) \end{matrix}$$

where *π****_α_***_0_ and *π****_β_***_0_ are the probabilities of *H*_01_ and *H*_10_, respectively. The equation makes an implicit assumption that the effects of ***α*** and ***β*** are independent of each other, such that the probability of ***α***=0 is not influenced by ***β***, and vice versa. This independence is guaranteed by the sequential ignorability assumptions ([Imai et al., 2010](#_ENREF_9)). DACT estimates *π****_α_***_0_ and *π****_β_***_0_ using novel methods that have been well-established in false discovery rate (FDR) literature, such as Efron’s approach using the central matching method ([Efron, 2004](#_ENREF_6), [Efron, 2007](#_ENREF_7)). Moreover, DACT has been shown to be comparable to or more powerful than existing methods across various simulation scenarios ([Liu et al., 2021](#_ENREF_10)).

# References

BARFIELD, R., SHEN, J., JUST, A. C., VOKONAS, P. S., SCHWARTZ, J., BACCARELLI, A. A., VANDERWEELE, T. J. & LIN, X. 2017. Testing for the indirect effect under the null for genome-wide mediation analyses. *Genetic Epidemiology,* 41**,** 824-833.

BERGER, R. L. & HSU, J. C. 1996. Bioequivalence trials, intersection-union tests and equivalence confidence sets. *Statistical Science,* 11**,** 283-319.

DAI, J. Y., STANFORD, J. L. & LEBLANC, M. 2022. A Multiple-Testing Procedure for High-Dimensional Mediation Hypotheses. *Journal of the American Statistical Association,* 117**,** 198-213.

DAVIES, R. B. 1980. Algorithm AS 155: The Distribution of a Linear Combination of chi-2 Random Variables. *Journal of the Royal Statistical Society: Series C (Applied Statistics),* 29**,** 323-333.

DJORDJILOVIĆ, V., PAGE, C., GRAN, J., N ST, T., SANDANGER, T., VEIER D, M. & THORESEN, M. 2019. Global test for high-dimensional mediation: Testing groups of potential mediators. *Statistics in medicine,* 38**,** 3346-3360.

EFRON, B. 2004. Large-Scale Simultaneous Hypothesis Testing. *Journal of the American Statistical Association,* 99**,** 96-104.

EFRON, B. 2007. Size, power and false discovery rates. *The Annals of Statistics,* 35**,** 1351-1377.

FRISCH, R. & WAUGH, F. V. 1933. Partial Time Regressions as Compared with Individual Trends. *Econometrica,* 1**,** 387-401.

IMAI, K., KEELE, L. & YAMAMOTO, T. 2010. Identification, inference and sensitivity analysis for causal mediation effects. *Statistical Science,* 25**,** 51-71.

LIU, Z., SHEN, J., BARFIELD, R., SCHWARTZ, J., BACCARELLI, A. A. & LIN, X. 2021. Large-Scale Hypothesis Testing for Causal Mediation Effects with Applications in Genome-wide Epigenetic Studies. *Journal of the American Statistical Association***,** 1-39.

LOVELL, M. C. 1963. Seasonal Adjustment of Economic Time Series and Multiple Regression Analysis. *Journal of the American Statistical Association,* 58**,** 993-1010.

MACKINNON, D. P., LOCKWOOD, C. M., HOFFMAN, J. M., WEST, S. G. & SHEETS, V. 2002. A comparison of methods to test mediation and other intervening variable effects. *Psychological Methods,* 7**,** 83-104.

QIAO, J., SHAO, Z., WU, Y., ZENG, P. & WANG, T. 2022. Detecting associated genes for complex traits shared across East Asian and Europeanpopulations under the framework of composite null hypothesis testing. *Journal of Translational Medicine*.

QU, L., GUENNEL, T. & MARSHALL, SCOTT L. 2013. Linear score tests for variance components in linear mixed models and applications to genetic association studies. *Biometrics,* 69**,** 883-892.

WU, M. C., LEE, S., CAI, T., LI, Y., BOEHNKE, M. & LIN, X. 2011. Rare-Variant Association Testing for Sequencing Data with the Sequence Kernel Association Test. *The American Journal of Human Genetics,* 89**,** 82-93.

ZENG, P., ZHAO, Y., LIU, J., LIU, L., ZHANG, L., WANG, T., HUANG, S. & CHEN, F. 2014. Likelihood Ratio Tests in Rare Variant Detection for Continuous Phenotypes. *Annals of Human Genetics,* 78**,** 320-332.
